# Passive acoustic monitoring of sound characteristics and vocalization patterns of the brown croaker

**DOI:** 10.1098/rsbl.2025.0314

**Published:** 2025-09-10

**Authors:** Hansoo Kim, Young Geul Yoon, Sungho Cho, Sunhyo Kim, Mira Kim, Donhyug Kang

**Affiliations:** ^1^ Sea Power Reinforcement·Security Research Department, Korea Institute of Ocean Science & Technology, Busan, Republic of Korea

**Keywords:** brown croaker, biological fish sounds, fish call and chorus, vocalization pattern, passive acoustic monitoring

## Abstract

Passive acoustic monitoring is an observation method for detecting and characterizing ocean soundscapes, and it has recently been used to observe underwater marine life. The brown croaker (*Miichthys miiuy*) is an important fish species in the Northwest Pacific Ocean that produces biological sounds. In this study, the sounds of 150 adult brown croakers were recorded continuously for three weeks using a self-recording hydrophone. The acoustic parameters of their calls, choruses and vocalization patterns were analysed using environmental factors from the ocean. The brown croaker’s call sound with zero peak sound pressure level was 150.8 dB, but the chorus sound was relatively high at 161.3 dB. The vocalization of the sounds occurred daily around sunset and dusk and was associated with decreased spawning activity when the water temperature decreased below approximately 25°C. The acoustic characteristics of the brown croaker’s sounds will help improve ocean soundscape management to protect the marine ecosystem and identify spawning and fishing grounds.

## 1. Introduction

In marine environments, numerous fish species can produce biological sounds associated with spawning and social communication [1–3]. The fish biophony within ocean soundscapes has been used to describe reproductive and spawning behaviours [4,5], identify essential fish habitats and analyse temporal and spatial distributions [6–8]. Passive acoustic monitoring (PAM) has recently been used in ocean soundscape research to study underwater sounds [9–11]. Long-term ocean soundscape monitoring can detect habitat changes over time, offering valuable insights for ecosystem monitoring and conservation research [12].

At least 178 fish families, accounting for two-thirds of all fish species, communicate using sound. They primarily use sound to court mates, defend food sources and territories and announce their presence to others [13,14]. The brown croaker (*Miichthys miiuy*) is a sound-producing benthic fish belonging to the Sciaenidae family that typically inhabits muddy or sandy bottoms at depths of 15−100 m and can grow over 1 m in length [15]. The brown croaker is primarily found in the East China Sea, the Yellow Sea and the South Sea of the Northwest Pacific Ocean. During the spawning season in summer, mature male brown croakers produce a wideband croaking sound while moving and to announce their presence to spawning females [16,17]. This sound is generated by the rapid contraction of the sonic muscle attached to the outer surface of the swim bladder. Variations in the shape and size of the swim bladder result in differences in the acoustic characteristics of the produced sound [18,19].

In this study, we aimed to elucidate the spawning characteristics of the brown croaker by analysing its acoustic signals using the PAM method. Little is known about the acoustic characteristics and vocalization patterns of the brown croaker in the ocean. We measured and analysed the species' call and chorus sounds and examined their correlation with daily variations, water temperature and fish vocalizations. We also explored the practical applications of these acoustic characteristics for monitoring the presence, abundance, and activity patterns of aquatic fish species.

## 2. Material and methods

Approximately 150 adult brown croakers (60–80 cm in total length) were housed and freely swum in a sea cage of the South Sea of Korea by our research institute’s Maritime Test and Evaluation Station (Tongyoung, 34°46′11.5″ N, 128°22′59.1″ E), affiliated with the Korea Institute of Ocean Science and Technology (KIOST) and their biological sounds were recorded and analysed.

An underwater self-recording hydrophone (SM3M, Wildlife Acoustics, Inc., USA) was deployed to continuously record acoustic data for 13 consecutive days (18–30 September 2023). A single hydrophone was used throughout the study. The hydrophone’s receiving voltage sensitivity was set to −164.6 dB V µPa^−1^, with a gain of 0 dB and a sampling frequency of 48 kHz. Brown croaker sounds were stored as digital files (*.wav) every 10 min.

To analyse the correlation between acoustic signals and marine environmental parameters, meteorological data, including sunrise and sunset times, were obtained from the Korea Meteorological Administration (https://www.weather.go.kr/w/index.do). At the same time, to understand the influence of biological sounds on ocean environmental variables, a water temperature sensor (RBR Duet T.D., RBR Global Inc., Canada) was installed alongside the hydrophone to record water temperature at a depth of 5 m at 10 s intervals.

Data processing and acoustic analysis were conducted using MATLAB (MathWorks, USA). The 13 days continuous underwater recordings contained numerous brown croaker vocalizations. The clipped signals were excluded, and a threshold level above the background noise was set to extract the brown croaker calls. For acoustic characteristic analysis, 150 representative call signals with a distinct pulse structure, no clipping and clear were selected.

The following parameters were analysed for call signals: call duration, pulses per call, inter-pulse interval (IPI), sound pressure level (SPL), peak frequency, minimum frequency, maximum frequency and −3 and −10 dB bandwidth. In contrast, only the SPL and peak frequency were analysed for chorus signals.

The call duration was determined as the mean time elapsed between the start and the end of the call signal, calculated based on the energy signal duration ratio, as defined by ISO 18405, and was characterized by the 95% cumulative energy point [20]. The IPI was calculated as the mean time elapsed between the end of one pulse and the beginning of the next consecutive pulse. Additionally, the SPL was analysed from the zero-to-peak (0–peak) signals [21–24]. Frequency analysis was performed using power spectral analysis for each independent signal duration. This analysis involved determining the peak frequency as the frequency component with the highest amplitude in the brown croaker’s sound. The −3 dB bandwidth corresponds to the range of frequencies that fall within 3 dB of the peak frequency amplitude, and the −10 dB bandwidth is the frequency range within 10 dB below the peak amplitude [22,24].

## 3. Results

**Figure 1.**
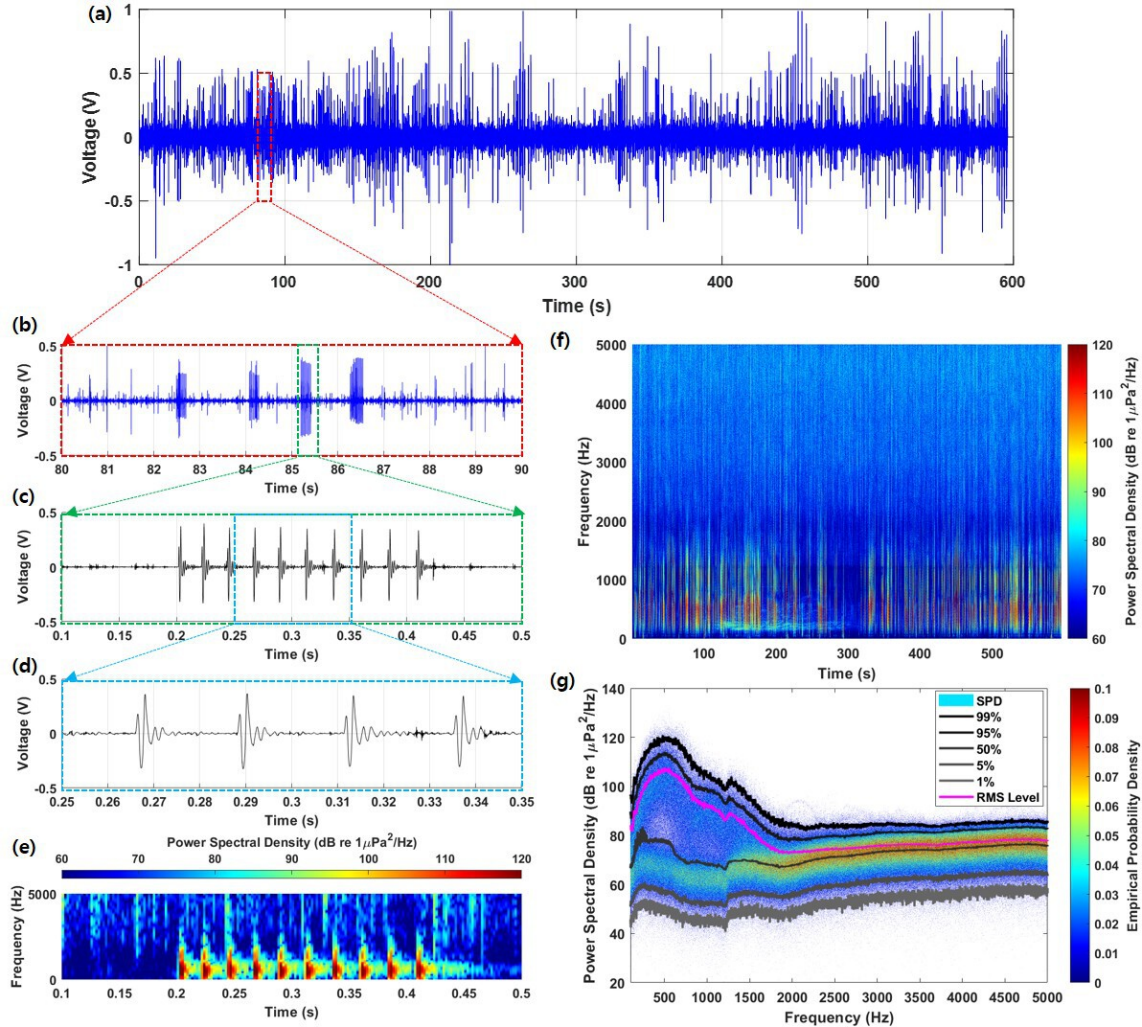
(a) Measuring acoustic data with a self-recording hydrophone for 10 min. (b) A close-up of the sounds of the brown croaker comprise four call sounds (boxed area shown in (a)). (c) A close-up of a time series of voltage signals corresponding to croaker sounds (boxed area shown in (b)). (d) A close-up of four pulses (boxed area shown in (c)). (e) Sound frequency spectrogram of croaker’s call. (f) Sound frequency spectrogram of croaker’s chorus. (g) Empirical probability density.

The recorded brown croaker calls consisted of multiple short, sequential pulses repeated throughout individual calls and choruses within the acoustic data (figure 1a,d). Each pulse signal featured low-amplitude positive and negative peaks, followed by a high-amplitude positive peak and single-peak decay.

The mean call duration was 119.9 ± 64.7 ms, with an IPI of 20.9 ± 2.5 ms and an average of 6.8 ± 3.6 pulses per call. The 0–peak mean SPL was 150.8 ± 2.4 dB. The peak frequency was 600.8 ± 117.5 Hz, while the minimum and maximum frequencies were 307.9 ± 102.7 and 905.2 ± 92.7 Hz, respectively. Spectral analysis revealed that −3 and −10 dB bandwidths corresponded to 199.1 ± 83.8 and 546.9 ± 197.7 Hz, respectively.

The root mean square level of the chorus exhibited the highest empirical probability density energy, primarily around 500 Hz, confirming that the brown croakers generated numerous call signals simultaneously, even within a 10 min recording period. During the observed chorus events, the 0–peak mean SPL was 161.3 dB and the peak frequency was 509.0 Hz (figure 1e,f). The detailed sound characteristics are summarized in table 1.

**Table 1.** Characterization statistics of brown croaker calls and choruses.

calls	*n*	mean ± s.d.	min.	max.
call duration (ms)	150	119.9 ± 64.7	23.1	257.5
inter-pulse interval (ms)		20.9 ± 2.5	17.5	23
pulse per call		6.8 ± 3.6	1	15
(0–peak) sound pressure level (dB)		150.8 ± 2.4	140.9	155.6
peak frequency (Hz)		600.8 ± 117.5	353	834
max. frequency (Hz)		905.2 ± 92.7	615	1214
min. frequency (Hz)		307.9 ± 102.7	135	586
−3 dB bandwidth (Hz)		199.1 ± 83.8	36	471
−10 dB bandwidth (Hz)		546.9 ± 197.7	276.5	1984

choruses	*n*	mean	—	—
(0–peak) sound pressure level (dB)	all observation data	161.3	—	—
peak frequency (Hz)		509.0	—	—

**Figure 2.**
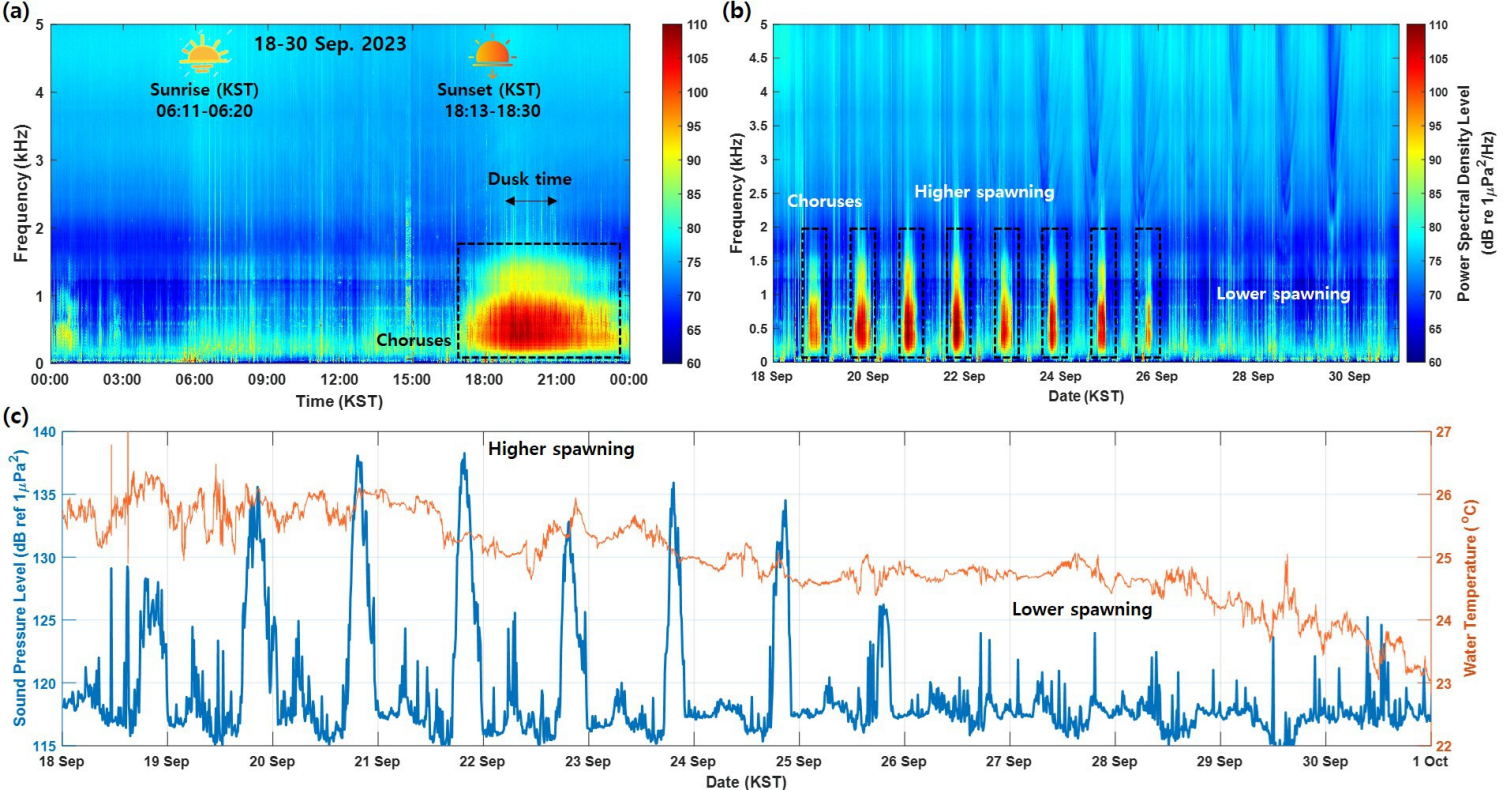
(a) Accumulated spectrogram of the daily variation in the choruses of the brown croaker. (b) Spectrogram of total observation days. (c) SPL and water temperature for total observation days.

All the observed signals were accumulated over 24 h to analyse daily variation patterns and are presented as a spectrogram up to 5 kHz (figure 2a). Although acoustic data were collected over 13 days, the local sunset time changed only slightly, from 18.30. to 18.13, with no significant impact on the duration of biological sounds. The vocalization of the brown croaker occurred daily around sunset and dusk, between 17.30 and 23.30 local time, and no spawning sounds occurred during dawn and daytime hours. The spawning-related acoustic energy was primarily concentrated at frequencies below 1 kHz, with additional effects observed below 1.6 kHz (figure 2a).

In cases where the brown croaker’s sounds were higher, spawning was observed in the evenings from 18 to 26 September 2023 (figure 2b). After this period, spawning sounds occurred at lower levels or were absent. The SPL associated with spawning activity decreased when the water temperature decreased below approximately 25°C (figure 2c).

## 4. Discussion

The acoustic properties of biological fish sounds, such as the brown croaker’s calls and choruses, generally increase during spawning. The spatial and temporal observations of brown croaker sounds recorded in this study using multiple self-recording hydrophones deployed in natural marine environments will help delineate spawning seasons and locations. Furthermore, these results provide fundamental data for investigating spawning behaviour and reproductive ecology.

Although some studies have examined the call sounds of brown croakers and other fish species of the Sciaenidae family [24–29], little is known about the acoustic characteristics of brown croaker calls in natural marine environments. Kim *et al.* [27] analysed the acoustic characteristics of brown croakers and their correlations with marine environmental factors [27]. However, their acoustic measurements were limited to a water tank environment, where reflections and scattering from the tank walls and bottom likely influenced the results. In the controlled indoor water tank environment, where a temperature chamber was used, the correlation between vocalization patterns, diel variation and water temperature differed from that observed in the natural marine environment, limiting the analysis. Ladich & Maiditsch [30] studied the acoustic characteristics of croaking sounds produced by croaking gouramis (*Trichopsis vittata*) while varying the water temperature to 25°C, 30°C and 35°C. Their findings demonstrated that acoustic communication in gourami fish is affected by ambient temperature changes [30]. Similarly, the brown croaker vocalization patterns are believed to be influenced by temperature fluctuations in both tank and natural marine environments. In previous research, brown croaker vocalizations were found to occur primarily between 11 and 25 October in water tank environments [27]. In contrast, vocalizations were most frequent in the natural marine environment between 18 and 26 September, highlighting differences in vocalization patterns. According to Lee *et al.* [31], the main spawning season for the brown croaker in the Korean coastal oceans is September [31], and Yoon *et al.* [32] reported that artificial spawning of the brown croaker is possible when the water temperature is above 25°C and salinity is about 30 ppt [32]. On the other hand, Moon *et al.* [33] simply reported that the spawning season and water temperature of the brown croaker were August to September and within 20.9−27.2°C through SST data, respectively [33]. In this study, the observations were conducted when the water temperature had already warmed, and it was confirmed that fish spawning sounds occurred above 25°C in the natural marine environment.

The time–frequency characteristics of the brown croaker’s calls estimated in this study were compared with the acoustic parameters of the Gulf corvina, Boeseman croaker and whitemouth croaker, all fishes of the Sciaenidae family known to produce biological sounds, as reported in previous studies (table 2) [24–26]. There were notable differences in the acoustic characteristics, such as the call duration, pulses per call and IPI, between the brown croaker and the other species. In particular, the call duration and IPI tended to increase. In contrast, the call duration seemed to decrease depending on the total length of the fish, Boesman croaker, Whitemouth croaker, brown croaker (in field condition), brown croaker (in tank condition) and Gulf corvina (in field condition) [24–26]. In addition, when only the brown croaker and Gulf corvina were compared, the peak frequency and −3 dB bandwidth tended to be lower, and the SPL tended to be higher with increased size.

**Table 2.** Comparative analysis of the habitats, ecological characteristics and acoustic parameters of call sounds among the Sciaenidae family.

information on Sciaenidae													
common name	brown croaker		Gulf corvina	Boeseman croaker	whitemouth croaker	Belanger’s croaker	large-eye croaker	big-snout croaker	donkey croaker	white croaker	big-head pennah croaker	Pawak croaker	tigertooth croaker
scientific name	*Miichthys miiuy*		*Cynoscion othonopterus*	*Boesemania microlepis*	*Micropogonias furnieri*	*Johnius belangerii*	*Johnius distinctus*	*Johnius macrorhynus*	*Pennahia aneus*	*Pennahia argentata*	*Pennahia macrocephalus*	*Pennahia pawak*	*Otoeithes ruber*
habitat distribution	Yellow & South Sea, East China Sea, Bohai Sea		Gulf of California Eastern Central Pacific	Indo-west Pacific	Mexico to the San Matías Gulf in Argentina	Indo-west Pacific	Northwest Pacific	Indo Pacific	Indo-west Pacific	Northwest Pacific	Indo-west Pacific	Western Pacific	Indo-west Pacific
habitat depth	15−100 m		1−30 m	10−100 m	1−120 m	~40 m	—	—	~60 m	20−140 m	~100 m	—	10−50 m
general size	60−90 cm		up to 100 cm	up to 100 cm	up to 90 cm	max. 30 cm	max. 22 cm	max. 30 cm	max. 30 cm	max. 40 cm	max. 27.8 cm	max. 23.2 cm	max. 90 cm
measurement length	60−80 cm	70−90 cm	unknown	average 40 cm	10−56 cm	unknown	unknown	unknown	unknown	unknown	unknown	unknown	unknown

acoustic parameters	mean ± s.d.		mean	mean ± s.d.	mean ± s.d.	mean ± s.d.	mean ± s.d.	mean ± s.d.	mean ± s.d.	mean ± s.d.	mean ± s.d.	mean ± s.d.	range
analysing no. call signals	150	411	139	10	112	200	242	85	90	104	92	169	17
call duration (ms)	119.9 ± 64.7	183.8 ± 27.4	389.2	108.2 ± 14.5		129.6 ± 44.6	132.5 ± 44.4	113.7 ± 32.0	119.4 ± 106.6	352.8 ± 242.7	247.5 ± 204.8	85.8 ± 54.5	—
pulses per call (times)	6.8 ± 3.6	8.9 ± 1.36	11.1	12.0 ± 1.6	—	10.2 ± 4.8	12.9 ± 3.8	11.2 ± 4.3	11.5 ± 8.7	11.4 ± 6.9	7.8 ± 5.5	9.2 ± 5.4	8−13
inter-pulse interval (ms)	20.9 ± 2.5	21.9 ± 1.0	32.6	8.7 ± 0.2	17.1 ± 5.81	6.4 ± 3.4	4.4 ± 1.9	3.9 ± 3.3	3.6 ± 1.7	25.6 ± 6.2	26.7 ± 7.4	2.9 ± 1.9	2.8−6.7
(0**–**peak) sound pressure level (dB)	150.8 ± 2.4	165.2 ± 0.7	176.3	—	—	—	—	—	—	—	—	—	—
peak frequency (Hz)	600.8 ± 117.5	459.2 ± 93.8	384.3	825	—	584 ± 181	511 ± 126	807 ± 143	736 ± 115	543 ± 98	576 ± 93	736 ± 101	354−1717
−3 dB bandwidth (Hz)	199.1 ± 83.8	79.1 ± 47.4	49	—	—	—	—	—	—	—	—	—	—
reference	this study	[27,34]	[24,34]	[25,34]	[26,34]	[28,29,34]							

Furthermore, a broader diversity of acoustic characteristics has been reported for Sciaenidae fishes [28,29], including call durations ranging from about 86 to 353 ms and peak frequencies ranging from about 354 to 1717 Hz. However, in most previous studies, fish size information was not provided alongside the recorded fish sounds, limiting the possibility of quantitative comparisons across croaker species.

The acoustic characteristics of the brown croaker offer valuable insights into its behavioural and reproductive ecology. The pronounced increase in vocalizations during spawning suggests that acoustic communication is crucial for coordinating reproductive activities and social interactions among individuals. The results of this study will benefit marine ecosystem conservation and the identification of spawning and fishing grounds.

## Data Availability

Data and figures are available from the Dryad Digital Repository [35].
